# The impact of nurse-led nonpharmacological multidisciplinary holistic nursing care on fatigue patients receiving hemodialysis: a randomized, parallel-group, controlled trial

**DOI:** 10.1186/s12912-022-01126-3

**Published:** 2022-12-12

**Authors:** Manhua Zuo, Wensheng Zhu, Jinrong Lin, Jing Zhuo, Xirui He, Xinghui Jing, Jun Tang, Renli Deng

**Affiliations:** 1 Department of Nursing, Zhuhai Campus of Zunyi Medical University, No. 368 Jinwan Road, Sanzao Town, Jinwan District, Zhuhai, 519041 China; 2 Dialysis center, Shanghai Hospital, No. 112 Shanghai Avenue, Wanzhou District, Chongqing, 445000 China; 3 Department of Foreign Languages, Zhuhai Campus of Zunyi Medical University, No.368 Jinwan Road, Sanzao Town, Jinwan District, Zhuhai, 519041 China; 4 Teaching department of humanities and social science, Zhuhai Campus of Zunyi Medical University, No.368 Jinwan Road, Jinwan District, Zhuhai, 519041 China; 5 Department of Bioengineering, Zhuhai Campus of Zunyi Medical University, No.368 Jinwan Road, Sanzao Town, Jinwan District, Zhuhai, 519041 China; 6 Department of Nephrology, the Fifth affiliated hospital, Zhuhai Campus of Zunyi Medical University, No.1439 Zhufeng Avenue, Doumen District, Zhuhai, 519100 China; 7 Nursing School of Zunyi Medical University, No. 6 Xuefu West Road, Xinpu New District, Zunyi, 563003 China

**Keywords:** Fatigue, Multidisciplinary, NICI, Nurse-led, Hemodialysis

## Abstract

**Background:**

Fatigue is a symptom characterized by an elevated prevalence in patients undergoing hemodialysis, which may cause extreme mental and muscular debilitation, significantly influencing social interaction, life quality and well-being. However, the significance of fatigue to patients undergoing hemodialysis has not been recognized yet, and prevention and management of fatigue in this population have not been thoroughly investigated. Additionally, previous studies mainly focused on muscular fatigue, while mental fatigue has been seldom discussed. This study aims to investigate the interaction between nurses and multidisciplinary of nonpharmacological integrated care interventions (NICIs) and assess the impact of fatigue on patients undergoing hemodialysis.

**Methods:**

The integrative nonpharmacological care interventions in this study included walking, motivational interviewing (MI) and health education regarding behavioral self-management. A single-center randomized controlled trial was conducted in the dialysis center of the nephrological department in a tertiary affiliated hospital of medical university from January to June 2019. A total of 118 patients were selected and randomly divided into the intervention group (IG) and the control group (CG). Four patients dropped out during the study, and 114 patients were enrolled for the eventual analysis. The 60 patients in the IG received routine nursing combined with integrated care interventions, while the 54 patients in the CG received routine nursing only. This study lasted for six months.

**Results:**

The experimental group exhibited significant reductions of overall fatigue (2.26 vs. 0.48), mental fatigue (1.41 vs. 0.54), muscular fatigue (2.13 vs. 0.75), and some biochemical indicators (e.g., serum urea) (*P*<0.05), compared with the CG.

**Conclusions:**

Nurses and multidisciplinary teams have been demonstrated to play a key role and interplay function in chronic disease management. Hence, the nurse-led multidisciplinary NICIs significantly alleviated total fatigue (muscular fatigue and mental fatigue) and improved other parameters.

**Trial registration:**

ChiCTR-IOR-16008621 (March 18, 2016)

## Background

Fatigue is a symptom characterized by an elevated prevalence (60~97%) in patients undergoing hemodialysis [1], which may lead to extreme mental and muscular debilitations, significantly influencing social interaction, life quality and well-being [2–4]. However, the significance of fatigue in patients undergoing hemodialysis remains unrecognized, and prevention and management of fatigue in hemodialysis have been seldom discussed [5, 6].

The self-management capability of patients undergoing hemodialysis is associated with dialysis compliance. However, low cognition level in the self-management of patients undergoing hemodialysis leads to suboptimal self-care such as limited dietary adherence, thus causing complications such as fatigue symptoms [7]. Meanwhile, education on self-management of health behaviors has been widely employed to facilitate management of patients with chronic diseases (e.g., diabetes mellitus) by sharpening cognitive self-management skills of these patients so that healthy behaviors can be developed [8].

Fatigue may cause inactivity and reduced physical functioning, which fosters a sedentary lifestyle and leads to degraded life quality [9]. Relevant authorities [10, 11] recommend 30-minute physical activity for patients with kidney disease (e.g., hemodialysis) almost daily. The effectiveness of physical activity on individual health has been demonstrated [12]. In particular, exercise programs, such as mild- to moderate-intensity walking, lead to improved fatigue symptoms and enhanced life quality of patients undergoing hemodialysis [13]. Hence, walking was incorporated as a lifestyle change in this study to relieve fatigue symptoms among patients undergoing hemodialysis.

Fatigue is typically divided as muscular fatigue and mental fatigue [9, 14]. To date, few services addressing mental fatigue in patients undergoing hemodialysis have been proposed, as psychological interventions are not possible without professionals with psychological expertise, while few nurses in the department of dialysis have qualifications in psychotherapy. Hence, an intervention requiring no specialized qualifications in psychology is urgently needed for patients undergoing hemodialysis with fatigue [15]. Recently, motivational interviewing (MI) conducted by personnel with no psychotherapist qualifications has been widely employed to tackle changes in various health behaviors of patients with chronic diseases [16]. Correspondingly, Helena *et al.* [17] reported that MI enhances dialysis adequacy and reduces the frequency of dialysis as well as serum levels of phosphorus (P) and albumin (ALB) by tuning the compliance of patients undergoing dialysis.

Previous studies on fatigue treatment focused on muscular fatigue instead of mental fatigue [18], and no medicine that can effectively prevent or treat fatigue of patients undergoing hemodialysis has been reported [19]. Interestingly, patients undergoing hemodialysis with fatigue prefer nonpharmacological interventions, which have been widely applied in patients with chronic diseases [20]. Additionally, multidisciplinary approaches are required for symptom management [18], and nurses may control fatigue by different methods [21].

This study aimed to investigate nurse-led multidisciplinary nonpharmacological integrated care interventions (NICIs) based on social, and psychophysiological factors, and the effects of nurse-led multidisciplinary NICIs on integrative fatigue and other indicators of patients undergoing hemodialysis compared with conventional nursing.

## Methods

A parallel-controlled, single-blind, and randomized trial was employed to evaluate the effectiveness of nurse-led NICIs specifically adapted for patients undergoing hemodialysis with fatigue. The trial was conducted in the dialysis center of the nephrological department in a tertiary affiliated hospital of medical university during the period of January 2019 to June 2019.

Inclusion criteria and exclusion criteria were identical to our previous publication [22]. Finally, a total of 118 patients were enrolled in this study and randomly allocated to either the intervention group (IG) or the control group (CG). Patients in the IG were administered with nurse-led nonpharmacological holistic care interventions (NHCIs) besides usual care, while patients in the CG were exposed to usual care only. A total of four patients (one quitted and one died in each group) were excluded during the clinical trial (no significant difference between the two groups), and 114 patients were ultimately analyzed after the six-month intervention.

The sample size was determined according to the mean of two samples. The primary outcome, as assessed by the RPFS, was fatigue. As reported, the numerical ranges of δ and σ^2^ are 1.05~2.10 and 2.11~3.43, respectively, indicating a significant difference. Based on a two-tailed test where the power was 0.8 and α was 0.05, the necessary total sample size was calculated to be 80~120. With practical circumstances taken into consideration, 100 was regarded as the critical sample size for this study, resulting in an attrition rate of 18%; eventually, 118 participants were enrolled, which satisfied the requirement.

At the implementation stage, the interventions were designed and executed by a team of experts with different expertise (eFigure 1). Before the main trial, the members of the intervention team, who were qualified, were trained on the interventions in three sessions for half a month. Participants took part voluntarily and signed informed consent, and the procedures were conducted accordingly. The IG was exposed to NHCIs, including health education in behavioral self-management [23], walking [13, 24] and MI [16, 17]. The NICIs were conducted based on the rationales of TOUS [25]. Education on self-management of health behaviors was delivered by means of simulations and audiovisual presentations, paper brochures, and spoken language. Daily walking steps were counted by using a pedometer (Meilen, eFigure 2), which was vertically hung from waistband/belt of the trousers (sleeping and bathing were an exception). During the entire period of this study, at least 6,000 steps daily were required for the participants. MI was applied in 20-minute sessions (once in a month). The standard care of the CG consisted of dieting, hydration, medication compliance, and health education, which is consistent with routine nursing care for patients undergoing hemodialysis. This study lasted for six months.

At the evaluation stage, fatigue was regarded as the primary outcome and it was assessed using the 22-item RPFS and a 10-point scale, which was identical to our previous publication [22].

The secondary indicators in this study were sociodemographic indicators, the vitality level of the SF-36 scale [26], the score of the HADS [27], the PSQI [28], the score of PSSS (Perceived Social Support Scale) [29] and the score of the Behavioral Self-Management Scale [30]. The SF-36 scale comprised of thirty-six items in eight dimensions (subscales) is administered [31]. The total score (range, 0~145) is calculated by using a previously reported method; the health status is proportional to the score [32]. Please refer to our previous publication for details about the secondary indicators [22].

### Data analysis

Continuous variables which obeyed normal distribution were presented as mean ± SD or median ± interquartile range in a skewed distribution; categorical variables were presented as frequencies and percentages. Inter- and intra-group differences of continuous variables were compared using independent t-tests or Mann–Whitney U tests. Inter- and intra-group differences of categorical variables were analyzed by using the Pearson’s chi-square test, McNemar’s test for related samples, or the McNemar-Bowker test of cross-tabulations. *P* < 0.05 indicated significant difference.

## Results

### Basic information of the studies

As illustrated in Fig. 1, a total of 216 patients were treated at the dialysis center of a local hospital during the six months. Among them, 98 refused to participate in this study and the remaining patients with fatigue and meeting inclusion criteria were enrolled and randomly divided into the IG (62) and the CG (56). Two patients in each group quitted before the end of this study. Eventually, the data of 114 participants were used for analysis.

### Individual and sociodemographic features of enrolled participants

45.61% of the participants were female, and 54.39% male (Table 1). The average age was 56.39±15.95 years. The incidence of fatigue in the CG and IG was 62.96% and 60.00%, respectively. 20 (33.33%) in the IG and 24 (44.44%) in the CG were aged 60 years or above. Other baseline parameters are also summarized in Table 1. No significant inter-group differences of these parameters were observed.

**Table 1:** Baseline characteristics of the two groups before intervention (categorical data, *n*=114)

Variable	Type	Overall sample	Experimental group	Control group	
		Frequency	Frequency	Frequency	*P*
Fatigue^**#^		70	36	34	0.863
Age (years)^**#^	≥60	44	20	24	0.175
Ethnicity^**#^	Han	42	23	19	0.851
Gender^**#^	Male	62	30	32	0.158
Comorbidities^**#^	≥3	45	23	22	0.275
Employment^**#^	Yes	5	3	2	0.886
Exercise^**#^	No	65	33	32	0.511
Exercise time^**#^	<30 min	85	43	42	0.226
Living situation^*#^	Alone	14	6	8	0.186
	Hybrid living	100	55	45	
Marital status^*#^	Separated	19	12	7	0.763
	Married	91	47	44	
	Divorced	4	2	2	
Education^**#^	Below elementary school	13	4	9	0.104
	Elementary school	29	15	14	
	Junior middle school	37	21	16	
	Senior High school or above	35	21	14	
Family income (RMB/month)^**#^	≤900	29	14	15	0.486
	901-1500	18	11	7	
	1501-3000	25	17	8	
	3001-5000	25	12	13	
	≥5001	17	8	9	
**Means of paying medical expenses** ^*#^	Own expense	6	3	3	0.127
	Medical insurance	53	31	22	
	Free medical service	4	3	1	
	Rural cooperative medical service	51	23	28	
Complications^**#^	Yes	95	51	44	0.275
Pain (whole body)^**#^	Yes	46	24	22	0.841
Pruritus^**#^	Yes	96	53	43	0.907
Appetite^**#^	Poor	48	24	24	0.592
	General	45	23	22	
	Normal	21	13	8	
Dialysis frequency (times/week)^*#^	1 time/week	4	0	4	0.068
	2 times/week	55	33	22	
	3 times/week	26	14	12	
	4 times/week	5	2	3	
	5 times/two weeks	24	13	11	

*Fisher’s exact test; **Pearson chi-square test; ^#^*P*>0.05

As shown in Table 2, the total fatigue scores of both groups were 5.82 vs. 5.89, while the severity of mental fatigue was higher than that of physical fatigue in both groups (6.27 vs. 5.21 and 6.35 vs. 5.31, respectively). Moreover, the self-management level of behaviors was 61.13 and 60.56 in the two groups, respectively, while the level of vitality was 13.07 and 13.49 in the IG and the CG, respectively. Additionally, clinical indicators, including the serum levels of calcium (2.10 vs. 2.10) and hemoglobin (100.83 vs. 102.90), ALB (39.13 vs. 39.25), were lower than the normal levels of individuals undergoing hemodialysis with fatigue.

**Table 2:** Comparison of pre-intervention baseline characteristics between groups (continuous data, *n*=114)

Indicators	Experimental group Mean (SD)	Control group Mean (SD)	***P***
**ALB (g/L)** ^**&**^	39.13(3.75)	39.25(4.95)	0.712
**Hb (g/L)** ^**&**^	103.21(22.09)	102.9(21.93)	0.458
**Fe (μmol/L)** ^**&**^	10.12(4.68)	10.04(5.37)	0.712
**TSAT**^**&**^	29.17(9.93)	29.62(11.36)	0.838
**P (mmol/L)** ^**&**^	2.05(0.50)	2.10(0.58)	0.055
**Ca (mmol/L)** ^**&**^	2.10(0.25)	2.10(0.25)	0.221
**Overall fatigue** ^**a&**^	5.82(1.57)	5.89(1.90)	0.854
**Mental fatigue** ^**&**^	6.27(1.93)	6.35(1.98)	0.882
**Muscular fatigue** ^**&**^	5.21(1.62)	5.31(2.15)	0.767
**PQSI** ^**d&**^	12.22(4.13)	11.93(3.64)	0.790
**Overall perceived social support** ^**b&**^	52.25(13.42)	53.69(13.37)	0.724
**Extrafamilial support** ^**&**^	28.94(12.18)	31.21(12.03)	0.521
**The vitality of SF-36** ^**c&**^	13.07(4.43)	13.49(3.90)	0.715
**Overall self-management behaviour** ^**f&**^	61.13(10.98)	60.56(10.22)	0.457
**Compliance with recommendations for liquid intake**^**&**^	12.47(4.21)	12.57(4.55)	0.928
**Depression** ^**e&**^	9.61(6.29)	10.90(4.85)	0.388
	Median, *P*25(*P*75)	Median, *P*25(*P*75)	
**Urea (mmol/L)** ^**#**^	8.45	10.00	0.235
	5.97(20.84)	6.87(20.09)	
**CRP (mg/L)**^**#**^	2.76	5.13	0.876
	0.54(13.06)	0.44(10.25)	
**PTH (pg/mL)** ^**#**^	392.09	288.29	0.531
	149.34(652.53)	168.89(492.98)	
**SF (μg/L)** ^**#**^	221.56	212.67	0.918
	54.62(686.87)	68.89(483.07)	
**Anxiety** ^**h#**^	3.00	2.50	0.341
	1.76(5.00)	0.24(4.74)	
**Intrafamilial support** ^**g#**^	23.00	23.00	0.568
	20.00(27.00)	21.00(23.00)	
**Self-monitoring disease** ^**#**^	5.00	5.00	0.563
	4.57(6.11)	5.14(7.57)	
**Protecting internal fistula** ^**#**^	3.50	4.50	0.276
	1.00(4.00)	2.15(4.00)	
**Compliance with recommendations for iron intake** ^**#**^	4.00 3.00(5.50)	3.00 3.00(5.00)	0.064
**Compliance with recommendations for sodium and protein intake** ^**#**^	14.50 10.74(17.00)	16.00 11.50(16.00)	0.512
**Developing good habits** ^**#**^	5.00	5.00	0.227
	52	5.00(5.39)	
**Compliance with medication regimen** ^**#**^	4.00 4.00(4.00)	4.00 4.00(4.00)	1.000
**Maintaining personal health** ^**#**^	5.00	4.50	0.476
	4.00(5.25)	3.55(4.50)	
**Seeking knowledge** ^**#**^	5.00	5.00	0.398
	2.00(7.00)	3.00(7.50)	
**Developing interests and hobbies** ^**#**^	1.00	1.00	0.283
	1.00(1.00)	1.00(1.00)	

Abbreviations: *Hb* haemoglobin; *ALB* albumin; *P* phosphorus; *Ca* calcium; *Fe* ferritin; *SF* serum ferritin; *TSAT* serum transferrin saturation; *SF-36* 36-Item Short-Form Health Survey; *PSQI* Pittsburgh Sleep Quality Index; CRP C-reactive protein*;* PTH parathyroid hormone; SF serum ferritin.

^a^ Total scores can range from 0 to 10, of which 0, 1–3, 4–6, and 7–10 indicate no symptoms, mild symptoms, moderate symptoms, and severe symptoms, respectively.

^b^ Total support scores can range from 12 to 84, with higher scores indicating better perceived social support.

^c^ Total scores can range from 0 to 145, with higher scores indicating better health.

^d^ Total scores can range from 0 to 21, with ≤ 7 points indicating normal sleep and > 7 points indicating a sleep disorder.

^e^ Total scores can range from 0 to 21, with < 7 points indicating no symptoms and ≥ 7 points indicating that symptoms are suspected or confirmed.

^f^ Total scores can range from 25 to 100, with a higher score indicating better behavioural self-management.

^g^ Intrafamilial support scores ranged from 4 28, with higher scores indicating better perceived social support.

^h^ Total scores can range from 0 to 21, with < 7 points indicating no symptoms and ≥ 7 points indicating that symptoms are suspected or confirmed.

^&^The variables were normally distributed and are expressed as the mean ± standard deviation (SD). Two-tailed independent-sample t-test, *P*>0.05.

^#^ The variables were non-normally distributed and are displayed as the median ± IQR, where the IQR is the difference between the 25th percentile (*P*25) and the 75th percentile (*P*75). Mann-Whitney U test, *P*>0.05.

### Effects of nurse-led NICIs within groups and between groups

As shown in Table 3, fatigue (physical, mental and overall), serum albumin, sleep disorders, vitality, anxiety, depression, compliance with liquid intake recommendations, compliance with iron intake recommendations, self-monitoring disease, maintenance of personal health, complications, pain (anywhere in the body) and appetite significantly varied in the IG (*P*=0.000), but not in the CG (*P*>0.05), after the intervention. In addition, fatigue (physical, mental and overall), self-management of behaviors, depression, sleep disorders, vitality, compliance with recommendations on intake of iron, liquid, protein, and sodium, TSAT, and urea of the two groups were significantly different after the intervention (*P*<0.05). Among these parameters, the variation of overall fatigue of the IG was significantly greater than that of the CG (*P*=0.000), and the variations of appetite were also significantly different after the intervention (*P*=0.025).

**Table 3:** Comparison of variables inter- and intra-group before and after six months of intervention (*n*=114)

Indicators	Experimental group (***n***=60)^**e**^		Control group (n=54)^**f**^		***P***
	Median, ***P***25(***P***75)		Median, ***P***25(***P***75)		
**TSAT**	8.05,2.27(14.89)		2.04,0.77(2.86)		0.000
**RPFS** ^**ae**^	2.26,1.45(3.44)		0.48,0.25(1.03)		0.000
**Mental fatigue** ^**e**^	1.41,0.61(2.89)		0.54,0.20(1.08)		0.000
**Muscular fatigue** ^**e**^	2.13,1.24(3.00)		0.75,0.22(1.16)		0.000
**PTH (pg/ml)**	22.64,-27.35(63.96)		8.38,-26.36(67.46)		0.653
**CRP (mg/L)**	1.83,-2.21(8.29)		1.30,-3.40(4.35)		0.211
**ALB (g/L)**^**e**^	6.15,1.11(8.32)		4.40,1.70(8.56)		0.643
**Hb (g/L)**	7.98,-1.99(27.96)		5.48,1.73(22.97)		0.734
**Fe (umol/L)**	3.17,-2.17(7.62)		0.74,-1.76(4.64)		0.184
**Ca (mmol/L)**	0.17,-0.05(0.45)		0.09,-0.10(0.28)		0.135
**P (mmol/L)**	0.31,-0.43(0.99)		-0.04,-0.32(0.27)		0.183
**SF (ug/L)**	25.34,-12.36(52.64)		10.63,-13.01(29.12)		0.289
**Urea (mmol/L)**	3.36,-0.06(11.74)		1.61,-3.44(5.93)		0.016
**PQSI** ^**c e**^	5.00,0.85(7.00)		2.00,0.00(2.00)		0.008
**Anxiety** ^**e**^	2.00,-1.00(3.00)		1.00,0.00(1.00)		0.616
**Vitality on the SF-36** ^**e**^	5.01,1.02(8.95)		1.00,1.00(4.20)		0.035
**Depression** ^**e**^	2.50,0.00(6.25)		0.00,0.00(0.00)		0.000
**PSSS** ^**b**^	6.06,2.78(6.04)		5.06,-16.08(21.05)		0.881
**Extrafamilial support**	2.12,-16.13(16.24)		2.16,0.09(2.06)		0.973
**Intrafamilial support**	4.11,2.05(4.08)		2.12,0.07(6.08)		0.343
**Overall behavioural self-management** ^**d**^	17.50,5.00(18.25)		5.50,1.00(10.00)		0.000
**Compliance with recommendations for liquid intake** ^**e**^	4.00,1.00(7.00)		1.00,0.00(3.20)		0.000
**Compliance with recommendations for sodium and protein intake** ^**e**^	3.00,1.00(5.00)		1.00,0.00(3.00)		0.000
**Compliance with recommendations for iron intake** ^**e**^	2.00,0.00(3.00)		0.00,0.00(0.00)		0.000
**Self-monitoring of disease** ^**e**^	1.00,0.00(2.00)		1.00,0.00(1.00)		0.164
**Protecting the internal fistula**	0.00,0.00(2.00)		0.00,0.00(1.00)		0.409
**Maintaining personal health** ^**e**^	1.00,0.00(1.00)		0.00, 0.00(1.00)		0.076
**Developing interests and hobbies**	0.00,0.00(1.00)		0.00, 0.00(0.00)		0.424
**Developing good habits**	0.00,0.00(1.00)		0.00,0.00(1.00)		0.337
**Seeking knowledge**	0.00, -1.00(2.00)		0.00, 0.00(1.00)		0.877
**Compliance with medication regimen**	0.00,0.00(0.00)		0.00, 0.00(0.00)		1.000
	Yes, n (%)	No, n (%)	Yes, n (%)	No, n (%)	
Complications*^#^	55(57.3)	5(27.8)	41(42.7)	13(72.2)	0.073
Pruritus*	50(53.2)	10(50.0)	44(46.8)	10(50.0)	0.909
Pain (whole body) *^@^	18(46.2)	42(56.0)	21(53.8)	33(44.0)	0.363
	Normal n(%)	General n(%)	Normal n(%)	General n(%)	
Appetite*^&^	10(62.5)	40(59.7)	6(37.5)	27(40.3)	0.029

Abbreviations: *CRP* C-reactive protein, *PTH* parathyroid hormone, *Hb* haemoglobin, *ALB* albumin, *P* phosphorus, *Ca* calcium; *Fe* ferritin, *SF* serum ferritin, *TSAT* serum transferrin saturation, *RPFS* Revised Piper Fatigue Scale; SF-36 36-Item Short-Form Health Survey; PSSS Perceived Social Support Scale; PSQI Pittsburgh Sleep Quality Index

The continuous within-group variables are reported as the median ± interquartile range of difference values between pre- and post-intervention measurements; the categorical variables are expressed as frequencies after intervention

^a^ Total fatigue scores can range from 0 to 10, of which 0, 1–3, 4–6, and 7–10 indicate no symptoms, mild symptoms, moderate symptoms, and severe symptoms, respectively

^b^ Total support scores can range from 12 to 84, with higher scores indicating better perceived social support

^c^ Total scores can range from 0 to 21, with ≤ 7 points indicating normal sleep and > 7 points indicating a sleep disorder

^d^ Total scores can range from 25 to 100, with a higher score indicating better behavioural self-management

^e^ Comparison of pre- and post-intervention values in the experimental group, *P*=0.000

^f^ Comparison of pre- and post-intervention values in the control group, *P*>0.05

*Data are reported as post-intervention values, and the two groups were compared using Pearson’s chi-square test

^#^ Comparison of pre- and post-intervention values in the experimental group by McNemar’s nonparametric test for related samples, *P*=0.000

^@^ Comparison of pre- and post-intervention values in the experimental group by McNemar’s nonparametric test for related samples, *P*=0.014

^&^ McNemar-Bowker test of cross-tabulations pre- and post-intervention in the experimental group, *P*=0.000

### Discussion

In this study, a controlled, randomized and parallel trial was performed to clarify the effectiveness of a nurse-led multidisciplinary NICI for patients undergoing hemodialysis with fatigue. As observed, the NICI significantly ameliorated total fatigue (including muscular fatigue and mental fatigue) and improved other parameters in patients undergoing hemodialysis with fatigue, similar to the previous findings [22]. This is also consistent with a previous study in which a combination of acupoint massages and aerobic exercise were significantly more effective in relieving carcinoma-associated fatigue and adjusting serum levels of phosphate compared with aerobic exercise alone [33].

Fatigue is one of the main stressors for hemodialysis patients, and a systematic review reported [34] that exercise interventions tend to be more effective in mitigating fatigue of adults receiving hemodialysis compared with routine care. Meanwhile, it has been demonstrated that patients undergoing hemodialysis may be demotivated from exercise training by the prospect of energetic activity. Hence, a less intensive exercise plan is needed for patients undergoing hemodialysis with fatigue [13]. The AASM guidelines indicate that indoor walking is a facile, low-cost, yet convenient physical activity for most patients. Therefore, walking, which is indeed an exercise training with mild intensity, was involved as part of the integrative interventions in this study.

Walking is also associated with improvement of mental functional status [13], which is consistent with the present study: the six-month mild-intensity walking training led to significantly improved muscular and mental fatigues. Meanwhile, walking may improve cardiac function and accelerate solute transport by enhancing tissue perfusion in a variety of organ systems, which accelerates large metabolite migration into the bloodstream and ultimately enhances dialysis to relieve symptoms, such as overall fatigue [35].

Self-management has a positive effect on patient health as it involves tasks such as medical management, role, and emotional domains [36]. As a critical effective care of chronic diseases, self-management involves daily health-related care functions [37]. One of the core elements of self-management is self-efficacy, which is associated with the capability of following recommended treatment and healthy behaviors [38]. A meta-analysis indicated a medium-sized positive effect of self-management on self-efficacy. However, self-efficacy also plays a meaningful role in improving self-care behaviors [39]. Likewise, another meta-analysis demonstrated that self-management of diet restrictions and fluid intake in patients undergoing hemodialysis had indirect control on the interdialytic weight gain, resulting in fatigue relief [40]. In this study, similar changes were observed. Specifically, the intervention led to enhanced self-management behaviors and mitigated overall fatigue, as well as effects on other indicators. The education on self-management in the present study aimed to relieve fatigue, reduce the disease-related cost, and enhance longevity and quality of life [41].

MI is a psychological intervention with no specialized psychological qualifications required. It aims to help individuals to overcome any ambivalence that may prevent them from adjusting their beliefs, as it is necessary to change behaviors (e.g., follow dietary advice) to improve life quality and relieve complications of the patients [42]. Hence, MI is indeed critical for prolonged disease management [43]. Specifically, MI induces cognitive changes, which in turn lead to behavior changes, including adhering to self-management protocols and a walking regimen. Ultimately, perceived bodily wellness is developed, as characterized by reductions of symptoms such as fatigue. This is consistent with systematic reviews and meta-analyses, which indicate that MI-based interventions were effective for increasing physical activity and improving adherence and communication, finally relieving numerous symptoms [44, 45]. Moreover, the self-efficacy element in self-management was associated with the initiative of changing lifestyle to achieve objectives [46], and this is consistent with the results of this study.

However, this study has several limitations: first, long-term follow-up was not involved after the intervention; second, the IG presumably attracted more attention by the staff compared with the CG; third, it was a single-center study, and the sample size was small; fourth, no quality control was involved for the process or the results of intervention.

## Conclusions

The NHCIs, which cannot be executed without a multidisciplinary team, are crucial and can improve various elements, including self-management skills, medication adherence and dietary compliance, facilitate self-management of behaviors and optimize clinical indicators. Meanwhile, the single role and interplay function of dialysis nurses and multidisciplinary teams in chronic disease management were verified. As a result, the effect of NICIs remain to be clarified by multicenter studies with follow-up. The biological mechanism of fatigue experienced by hemodialysis patients and the role of specific indicators in NICIs for patients undergoing hemodialysis with fatigue remain to be further investigated in the future.

## Data Availability

The datasets used and/or analyzed during the current study are available from the corresponding author (MH ZUO) upon reasonable request.

## Abbreviations

MI: motivational interviewing
RPFS: Revised Piper Fatigue Scale
Hb: haemoglobin
ALB: albumin
P: phosphorus
Ca: calcium
Fe: ferritin
SF: serum ferritin
TSAT: serum transferrin saturation
SF-36: 36-Item Short-Form Health Survey
PSQI: Pittsburgh Sleep Quality Index
PSSS: Perceived Social Support Scale
CRP: C-reactive protein
PTH: parathyroid hormone
SF: serum ferritin
